# Robotic systems for upper-limb rehabilitation in multiple sclerosis: a SWOT analysis and the synergies with virtual and augmented environments

**DOI:** 10.3389/frobt.2024.1335147

**Published:** 2024-02-27

**Authors:** Giulia A. Albanese, Anna Bucchieri, Jessica Podda, Andrea Tacchino, Stefano Buccelli, Elena De Momi, Matteo Laffranchi, Kailynn Mannella, Michael W. R. Holmes, Jacopo Zenzeri, Lorenzo De Michieli, Giampaolo Brichetto, Giacinto Barresi

**Affiliations:** ^1^ ReWing s.r.l., Milan, Italy; ^2^ Rehab Technologies Lab, Istituto Italiano di Tecnologia, Genoa, Italy; ^3^ Department of Electronics, Information and Bioengineering, Politecnico di Milano, Milan, Italy; ^4^ Scientific Research Area, Italian Multiple Sclerosis Foundation (FISM), Genoa, Italy; ^5^ Department of Kinesiology, Brock University, St. Catharines, ON, Canada; ^6^ AISM Rehabilitation Center Liguria, Italian Multiple Sclerosis Society (AISM), Genoa, Italy

**Keywords:** multiple sclerosis, robotics, rehabilitation, SWOT, virtual reality, augmented reality, digital health

## Abstract

The robotics discipline is exploring precise and versatile solutions for upper-limb rehabilitation in Multiple Sclerosis (MS). People with MS can greatly benefit from robotic systems to help combat the complexities of this disease, which can impair the ability to perform activities of daily living (ADLs). In order to present the potential and the limitations of smart mechatronic devices in the mentioned clinical domain, this review is structured to propose a concise SWOT (Strengths, Weaknesses, Opportunities, and Threats) Analysis of robotic rehabilitation in MS. Through the SWOT Analysis, a method mostly adopted in business management, this paper addresses both internal and external factors that can promote or hinder the adoption of upper-limb rehabilitation robots in MS. Subsequently, it discusses how the synergy with another category of interaction technologies - the systems underlying virtual and augmented environments - may empower Strengths, overcome Weaknesses, expand Opportunities, and handle Threats in rehabilitation robotics for MS. The impactful adaptability of these digital settings (extensively used in rehabilitation for MS, even to approach ADL-like tasks in safe simulated contexts) is the main reason for presenting this approach to face the critical issues of the aforementioned SWOT Analysis. This methodological proposal aims at paving the way for devising further synergistic strategies based on the integration of medical robotic devices with other promising technologies to help upper-limb functional recovery in MS.

## 1 Introduction

The extraordinary growth of robotic applications to rehabilitation has offered multiple solutions for the most demanding issues of people with disabilities (Carbone and Gonçalves, 2022; Pierella and Micera, 2022; Sadeghnejad et al., 2023). Among these challenges, we can find the impairments caused by Multiple Sclerosis (MS), a complex disorder of the central nervous system, showing a spectrum of sensory, motor, autonomic, and cognitive difficulties that severely affect a person’s capability to perform several Activities of Daily Living (ADLs) (Dobson and Giovannoni, 2019; Lublin et al., 2022). The signs and symptoms of MS are the consequence of underlying neuropathologic changes that occur in the central nervous system (CNS). The primary mechanism of injury is inflammatory demyelination and, to a variable degree, axonal damage (Lublin, 2005). The classification of MS into subtypes plays a crucial role in both prognosis and treatment decisions. The four subtypes of MS, namely, Relapsing-Remitting MS (RRMS), Primary Progressive MS (PPMS), Secondary Progressive MS (SPMS), and Progressive-Relapsing MS (PRMS), are characterized by distinct clinical manifestations (Lublin et al., 2014; Giovannoni et al., 2016). During RRMS, inflammatory attacks on myelin and nerve fibers cause visual impairments, tingling and numbness, fatigue, intestinal and urinary system disorders, spasticity, and learning and memory impairment. PPMS mainly affects the nerves of the spinal cord, leading to walking difficulties, weakness, stiffness, and balance problems. SPMS is considered the second phase of RRMS and affects around 65% of patients, causing increased weakness, fatigue, stiffness, mental disorders, and psychological impairment. PRMS is the rarest type of MS and affects approximately 5% of patients, presenting symptoms like eye pain, double vision, sexual, intestinal, and urinary system dysfunction, dizziness, and depression (Ghasemi et al., 2017).

Roboticists explore the potential of smart mechatronic devices in the domain of MS by approaching the inter-individual variability and unpredictable progression of the disease to provide patients with dynamic and personalized approaches to rehabilitation (Lamers et al., 2018; Duan et al., 2023; Podda et al., 2023). By offering precise and versatile tools to clinicians, the field of biomedical robotics contributes to both studying and treating this multifaceted disease (Rajavenkatanarayanan et al., 2019), especially in terms of motor impairments due to symptoms like muscle weakness, spasticity, fatigue, tremors, coordination difficulties, and deficits in postural and motion control. We should also ponder how motor-cognitive impairments are a priority that is also targeted by the developers of robots for rehabilitation in MS. However, barriers to the introduction of these solutions in clinical settings exist, especially considering the specific symptoms of the disease (e.g., spasticity could exclude the use of devices mechanically acting on the individual’s limbs for assisting the recovery of other skills, like cognitive ones) and the cost of purchasing the devices. Nevertheless, opportunities in this domain definitely exist alongside the potential for responses to the aforementioned challenges. The recent reviews by Straudi et al. (2022) and Dixit and Tedla (2019) have highlighted the need for additional high-quality trials with sufficient sample sizes and methodological rigor to draw definitive conclusions about the effectiveness of the clinical application of robotic-assisted upper limb-therapy in MS. This manuscript explores upper-limb robotic rehabilitation in MS through a SWOT analysis (Strengths, Weaknesses, Opportunities, and Threats) (Rizzo and Kim, 2005; Nwosu et al., 2019). The objective of this review is to exploit this approach to elucidate the potential of robotics in MS rehabilitation by providing a comprehensive perspective and addressing future research in meeting the evolving needs of the field. While conventionally employed to assess factors influencing a company’s competitive position, SWOT analysis transcends the business realm and can be employed in diverse fields. Rizzo and Kim (2005) has served as a precedent of prior use of SWOT analysis in Virtual Rehabilitation and Therapy, highlighting the versatility of this framework beyond traditional business applications. Essentially, this framework assists in planning and organizing any human endeavors, helping find the internal strengths and weaknesses and the external trends (opportunities and threats) faced by the entity. This approach aimed to stimulate the proposal of innovative solutions able to exploit identified strengths, address acknowledged weaknesses, capitalize on available opportunities, and mitigate potential threats. Applied to our context, the SWOT analysis wanted to be a methodological contribution aimed at proposing a business-oriented perspective that can support a patient-centered approach, taking into consideration market access issues as well. Additionally, this discussion is integrated with a debate on how the synergy between robotic devices and interactive systems, particularly virtual and augmented settings, might be a solution to enhance the hidden potential of robotic rehabilitation alone for People with MS (PwMS). The potential of virtual/augmented systems in engaging PwMS, and also providing clinicians with adaptable options for improving treatments, is quite well-known in the literature (Calabrò et al., 2017; Russo et al., 2018). The choice of analyzing if these solutions can move upper-limb rehabilitation robotics in MS beyond its state-of-the-art is an example we present to the community of researchers, developers, clinicians, patients, and all stakeholders. We expect that other solutions can be explored, obviously, according to the framework we propose here. On the other hand, this choice will be fully elucidated after the first two sections. We will commence by exploring the features of robot-based upper-limb rehabilitation in MS, followed by a comprehensive SWOT analysis of these solutions. Subsequently, we will delve into the world of virtual/augmented systems for rehabilitation in PwMS and examine the synergies between these latter and robotic devices, discussing their transformative potential and impact.

## 2 Robot-based upper-limb rehabilitation for PwMS

Robotic devices have been increasingly used in neurological rehabilitation due to their ability to provide repetitive and highly reproducible motor movements, leading to positive results in motor learning and in the development or restoration of motor pathways (Krebs et al., 2007; Vergaro et al., 2010; Lo and Xie, 2012). Besides allowing for intensive training, these technologies offer the opportunity to measure real-time performance and assess the sensorimotor function of one’s limb (Iandolo et al., 2019). In the context of MS rehabilitation, several studies have investigated the use of different upper-limb robotic devices, including both research prototypes and commercial devices.

The search for relevant studies was conducted in PUBMED, SCOPUS, IEEE, Cochrane Library, Physiotherapy Evidence Database (PEDro), on papers published up to May 2023. To ensure the inclusion of pertinent literature, we established specific criteria to guide our selection process: in the title, abstract and keywords, we looked for rehabilitat* AND multiple sclerosis AND robot*/exoskeleton/end-effector/haptic device AND upper/hand/arm/wrist/fingers. In this manuscript, we considered the terms “robot” and “haptic interface” as interchangeable to refer to a robotic device that is used to guide, perturb, or restrict the movements of a person in direct contact with the robot’s end effector (Harwin et al., 2006). We excluded any review and all those publications in which either robotic devices were used only for the assessment of upper-limb function in MS or no person with MS have been tested. This approach allowed us to identify 19 publications that met our criteria. Among these, two papers (Tramontano et al., 2020a; Tramontano et al., 2020b) were excluded. Indeed, although the authors defined the training protocol as “robot-based rehabilitation” (Tramontano et al., 2020b), the system involved there was Pablo, which is a sensor-based technology that lacks any controlled haptic feedback. Therefore, we deemed it inappropriate for inclusion in our manuscript. The resulting list of papers can be found in Table 1, organized according to the device employed in the study. The devices, which are listed and briefly described in Section 2.1, include two research prototypes (Braccio di Ferro and Wristbot) and four commercialized robots. All of them can employ haptic feedback to simulate a diverse and adaptable environment, incorporating visual, auditory, kinesthetic, and proprioceptive stimulations.

**TABLE 1:** Publications organized according to the robotic devices used.

Robotic device	Publications
Braccio di Ferro	Vergaro et al. (2010); Carpinella et al. (2009, 2012); Solaro et al. (2020); Basteris et al. (2011); Groppo et al. (2017)
Wristbot	Mannella et al. (2021)
Armeo Spring	Gijbels et al. (2011); Sampson et al. (2016); Manuli et al. (2020b)
Haptic Master	Feys et al. (2015); Maris et al. (2018); Octavia and Coninx (2014); Tedesco Triccas et al. (2022)
Phantom	Feys et al. (2009); Xydas and Louca (2012)
Amadeo	Gandolfi et al. (2018)

Overall, PwMS have provided positive feedback about the use of robotic devices and reported high scores regarding the perception of motor and mental wellbeing after undergoing customized training (Manuli et al., 2020b). Indeed, motivation is a crucial predictor of treatment success (Grahn et al., 2000), and the use of robotic devices in rehabilitation has been shown to be an attractive tool that allows users to embrace a positive attitude without feeling stressed or pressured.

**FIGURE 1:**
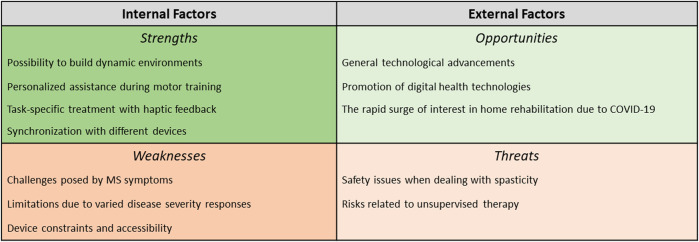
Summary of SWOT analysis for robotic rehabilitation in PwMS.

After briefly describing the robotic systems used for upper limb rehabilitation in PwMS (Section 2.1), a SWOT analysis will be employed to investigate the use of robotic devices for upper-limb rehabilitation in PwMS. The following sections will describe and discuss the emergence of Strengths and Weaknesses of robotic technologies for rehabilitation as evidenced by research in the field. The second half of the SWOT analysis will provide readers with possible future Opportunities and Threats that are emerging from external factors and developments in related fields. Figure 1 summarizes the SWOT analysis presented in Sections 2.2–2.5.

### 2.1 Robotic systems used for upper limb rehabilitation in PwMS

1. Braccio di Ferro (Casadio et al., 2006) is a haptic planar manipulandum with 2 Degrees of Freedom (DoFs). The robotic device has a rigid structure and two brushless motors that offer full back-drivability, and low intrinsic mechanical impedance. The rotations of the shoulder and elbow determine and are determined by the kinematics of the hand, which grasps the device through a handle.
2. Wristbot (Iandolo et al., 2019) is a manipulandum that allows 3-DoF wrist rotations in a human-like range of motion. Grasping the handle of the device, the Wristbot assures low inertia and gravity compensation during the user’s active motion, but it is also able to provide the torques needed to manipulate the wrist joint during passive or assistive modalities.
3. The Armeo Spring (Hocoma AG, Volketswil, CH) (Gijbels et al., 2011) is a 5-DoF (3 in the shoulder, 1 in the elbow, 1 in the forearm) orthosis without robotic actuators. The adjustable mechanical arm allows variable levels of gravity support by a spring mechanism, that enables users with residual upper limb function to achieve a larger active range of motion (ROM) within a 3-dimensional workspace.
4. The Haptic Master (MOOG, Nieuw-Vennep, NL) (Feys et al., 2015) is a commercially available end-effector-based robot that allows 3-dimensional movements with 6-DoFs. Three actuated DoFs are for positioning and three non-actuated DoFs are for orientation in the gimbal. This configuration permits the person to freely orient, open and close their hand as needed to manipulate an object.
5. The Phantom (SensAble Technologies Inc., MA, United States) (Feys et al., 2009) is an end-effector haptic device, controlled by 3 motors. It is handled through a pen-like stylus which provides force feedback in 3-DoFs. Unrestricted movements of the shoulder, elbow, and wrist joints are possibly involved during its use.
6. Amadeo (Tyromotion GmbH) (Gandolfi et al., 2018) is a 5-DoF device for hand rehabilitation. Amadeo can provide position-based passive, active, and assistive training modes, centered on the flexion and extension of each finger. The moving finger slides are attached to the fingers using a small magnetic disc and adhesive tape for connection to the robot. The slides then transfer, bending or stretching, movements to the fingers.

### 2.2 Strengths

#### 2.2.1 Possibility to build dynamic environments

A prerequisite for both robot- and therapist-assisted rehabilitation is that individuals must maintain their ability to adapt to new dynamic environments (Shadmehr and Mussa-Ivaldi, 1994). Indeed, implicit motor adaptation may be able to reshape the altered sensorimotor mappings and contribute to cortical reorganization, potentially limiting the consequences of irreversible tissue damage in normal-appearing brain tissue and MS lesions (Reinkensmeyer et al., 2004; Rocca et al., 2005). For this reason, adaptive training protocols that introduce unfamiliar dynamic environments for individuals to adapt to, rather than simply assisting them during movement practice, may be beneficial to PwMS (Patton and Mussa-Ivaldi, 2004). Although PwMS have demonstrated residual capabilities for sensorimotor adaptation in arm and posture control, cerebellar deficits have been linked to difficulties in adapting to novel dynamic environments (Maschke et al., 2004; Smith and Shadmehr, 2005). It has been shown that individuals with cerebellar degeneration lose the motor learning mechanism based on the feed-forward control component and involved in motor adaptation (Maschke et al., 2004; Smith and Shadmehr, 2005). Individuals with MS still display this mechanism, albeit impaired (Leocani et al., 2007; Casadio et al., 2008) in such a way that could contribute to the coordination deficit and tremor associated with MS. Since force field adaptation exercises can train this feed-forward control mechanism, they may be effective in reducing tremor, improving upper limb coordination, and reducing disability in PwMS. Adaptive training may, therefore, be a promising rehabilitation approach for PwMS who exhibit various types and degrees of deficits. In the literature, this approach based on targeting sensorimotor adaptation in dynamic environments can be found in some protocols tested on PwMS (Carpinella et al., 2009; Vergaro et al., 2010; Basteris et al., 2011; Solaro et al., 2020). All these cited studies employed the Braccio di Ferro (Casadio et al., 2006) to develop an 8-session-long rehabilitative protocol involving robot-based reaching movements. In some of these studies (Carpinella et al., 2009; Basteris et al., 2011; Solaro et al., 2020), the task consisted of a series of reaching movements with a position-dependent resistive force directed along the line that connected the end-effector to the target, designed to challenge muscle weakness. However, the instability of the environment was given by additional force fields: a velocity-dependent force perpendicular to the instantaneous movement direction in Carpinella et al. (2009), and a virtual point mass connected to the subjects’ hand through a linear spring, that acted as “virtual tool”, in the remaining studies (Basteris et al., 2011; Solaro et al., 2020). Differently, in the protocol of Vergaro et al. (2010), by means of an iterative procedure, the robot learned the forces necessary to generate a perturbation directed orthogonally with respect to the trajectory that, for each target direction, either enhanced or decreased the lateral deviation of the average trajectories of the subject, estimated during a baseline session. Indeed, the procedure used in some works (Vergaro et al., 2010; Basteris et al., 2011; Solaro et al., 2020) was to calculate forces and spring stiffness at the beginning of each session and to let the protocol adapt its difficulty to the subject’s specific impairment and the improvements - if any - that occurred from session to session. The results of these works showed that PwMS revealed a preserved ability to adapt to robot-generated forces, greater in subjects with non-cerebellar symptoms (Solaro et al., 2020). In particular, subjects showed smoother and more linear movements (Vergaro et al., 2010; Solaro et al., 2020) over and within sessions. In addition, several studies have reported a significant improvement in the Nine-Hole Peg Test (9HPT) following training (Carpinella et al., 2009; Vergaro et al., 2010; Basteris et al., 2011; Solaro et al., 2020), indicating a potential transfer of therapy benefits to activities of daily living. Although the 9HPT primarily assesses manual dexterity, it requires coordination of the entire limb. Therefore, even though when using Braccio di Ferro the hand is not actively involved in the reaching exercise, the improvement observed in the 9HPT may be linked to enhanced coordination in the elbow and shoulder.

#### 2.2.2 Personalized assistance during motor training

Robotic devices are capable of providing haptic feedback in a controlled manner, not only to perturb the environment but also to assist in movement execution. Given the wide variety of symptoms and their different severity in PwMS (Lublin et al., 2014), assistance should be tailored according to the motor skills of the individual (Casadio and Sanguineti, 2012; Gassert and Dietz, 2018). Assistance can take the form of gravity support, guidance through specific movement patterns, or help with movement completion. A concern when providing excessive assistance is the “Slacking” effect (Casadio and Sanguineti, 2012), which refers to a reduction in voluntary movement control caused by repetitive passive mobilization of the limbs. To avoid this, a potential solution is to implement the real-time tailoring of the assistance according to the individual’s needs and actual abilities. This “assistance-as-needed” approach seeks to reduce the risk of patients becoming overly reliant on robotic assistance, which could decrease their level of participation and hinder the potential for neuroplastic changes (Wolbrecht et al., 2008). Robot-based personalized assistance has been used with PwMS in several studies (Xydas and Louca, 2012; Groppo et al., 2017; Mannella et al., 2021) employing the Braccio di Ferro (Casadio et al., 2006), the Wristbot (Iandolo et al., 2019) and on an end-effector haptic device handled through a pen-like stylus comparable to the PHANTOM (Xydas and Louca, 2012). The study of Groppo et al. (2017) proposed a 23-session-long protocol to deal with the progressive worsening of motor functions in one PwMS. This multidisciplinary protocol involved traditional occupational therapy and a robot-based task, during which the subject performed center-out reaching movements. After 2 s from the movement onset, unless the subject was able to reach the target on their own, a minimally assistive force modulated according to the hand speed was generated by the robot. Groppo et al. (2017) found signs of improved motor control, given the significant increase in both the velocity and the smoothness of arm trajectories during robot-based reaching movements. Additionally, the fMRI revealed that multidisciplinary rehabilitation in MS seems to be clinically efficacious and to have a significant impact on brain functional reorganization in the short-term (Groppo et al., 2017). Mannella et al. (2021) trained the most affected limb of 7 PwMS in a 4-week robot-based program. The task was a continuous tracking of a figure targeting continuous movements in the flexion/extension and radial/ulnar deviation 2-dimensional space, in the presence of an assistive force. The force was implemented as a spring pulling the subject toward the target, and its rigidity was modulated within and between sessions, according to the performance of the subject in terms of accuracy in tracing the path. Actually, when the performance reached a specific level, assistance switched to resistance and pushed the subject far from the target, thus generating a dynamic environment to which to adapt. Similarly to what was found by Groppo et al. (2017), at the end of the treatment period, the authors detected a greater motor accuracy and control (Mannella et al., 2021), quantified by lower errors in tracking and tracing the target. In contrast, Xydas and Louca (Xydas and Louca, 2012) proposed an augmented version of the 9HPT, which incorporates assistive forces to transform it into a physiotherapy and rehabilitation system. The system included adaptive assistive forces based on healthy users’ reference target trajectories and was evaluated in a single session by three PwMS. The results showed a potential improvement in the upper limb performance in 3-dimensional reaching tasks, indicating that the system could be effectively used for rehabilitation in complex movements. Analogously to the personalization of the level of assistance, Octavia and Coninx (2014) adapted the difficulty level of the training tasks proposed. Based on the information about the training progress of the subject, the algorithm determined if and how the difficulty level should have been adapted. The study revealed that the participants followed different training patterns and progression, thus confirming the need for personalized levels of difficulty. This adaptive personalized training has shown to be beneficial and appreciated by users (Octavia and Coninx, 2014).

#### 2.2.3 Task-specific treatment with haptic feedback

Robotics allows for repetitive and consistent motor movements at high dosages, however, repetition alone, without usefulness or meaning in terms of function, is not enough to produce increased motor cortical representations (Bayona et al., 2005). Rather than on the specific impairment, rehabilitation should focus on task-specific training to improve the performance in functional tasks through goal-directed practice and repetition. Task-specific training means practicing context-specific motor tasks while receiving some form of feedback (Schmidt and Lee, 1988). Robot-aided haptic feedback is a promising approach for rehabilitation, as it can provide information to supplement or substitute visual and auditory cues (Demain et al., 2013). This kind of feedback can help internalize the movements and increase proprioceptive awareness, thereby enhancing motor learning (Winter et al., 2022). Motor learning and skill acquisition are able to elicit the functional reorganization of cortical areas and the development of new motor pathways to restore limb function (Plautz et al., 2000). The Haptic Master has been used by a few studies (Octavia and Coninx, 2014; Feys et al., 2015; Maris et al., 2018) to train PwMS in an 8-session-long robot-based protocol additional to the conventional therapy. All exercises required accurate and stabilized end-positions to successfully perform the task-oriented movements. The exercises varied in the number of movement directions (1-2-3D), the haptic environment, the precision level and type of required movements, and the cognitive load. At the end of the training, movement tasks in three dimensions, measured with the robot, were performed in less time and more efficiently (Feys et al., 2015). Significant improvements were found in Maris et al. (2018) for shoulder ROM, handgrip strength, perceived strength, and Wolf Motor Function Test (WFMT) activities. Tedesco Triccas et al. (2022) investigated the impact of the intervention of Maris et al. (2018) on patients’ lives: 1) Participants felt that there was a positive impact of the training on strength, endurance, and during activities of daily living; 2) Participants expressed feelings of motivation and self-improvement about the system usage. Similarly, Gijbels et al. (2011) employed the Armeo Spring system to develop a mechanical-assisted therapy involving repetitive and active exertion of goal-directed movements, during the practice of complex motor tasks. The 8-week-long protocol, additional to conventional therapy, included the repetition of 5 tasks, ranging from gross movement, over more precise movement, to subtle strength-dosed movement. Significant gains were found in functional capacity tests [Upper extremity performance test for the elderly (TEMPA), 9HPT, Action Research Arm Test (ARAT)], particularly in subjects whose upper limb function was mostly affected at baseline. Gandolfi et al. (2018) compared two approaches for a 5-week-long training: a robot-assisted hand training using Amadeo that was mainly focused on visual feedback and had a task-specific approach, and a robot-unassisted training that dealt with functional movement and context-specific training. Both training protocols shared common features such as unilateral training, mobility, stretching, and exercise progression. However, only the robotic training involved more intensive, repetitive, and task-specific exercises. The main finding of this study was that upper limb activity and function improved after both treatments but only the robot-assisted hand training reported significant improvements in the assessment of skills in the life habits domain (Motor Activity Log (Taub et al., 1993)). In addition, preliminary observation of muscular activity showed enhancement of the extensor carpi radialis activation only in the robot-assisted group, suggesting a task-specific effect of this training mode on muscle activity. Finally, Feys et al. (2015) tried to investigate which types of robotic outcome measures could be clinically relevant. The protocol lasted 4 weeks and involved tasks that required motor accuracy, ROM, and the ability to exert high-speed movements, with different levels of assistance and difficulty. Significant correlations were found between specific functional measures (specifically ARAT and Purdue pegboard test) and movement tasks.

#### 2.2.4 Synchronization with different devices

Robotic devices offer the opportunity to be synchronized with other electronic systems, in order to assess a broader set of body signals (Rizzoglio et al., 2020), deliver external additive feedback (Cuppone et al., 2016) and apply therapeutic stimulations (Sampson et al., 2016). The core instances of systems synchronized to robots are screen displays on which visual feedback of the virtual reality environment associated with the task is provided. Apart from these, it is hard to find examples of the use of devices in combination with robots for the rehabilitation of PwMS. The only study that apply was conducted by Sampson et al. (2016), combining the Armeo Spring (Sanchez et al., 2004) with Functional Electrical Stimulation (FES). The protocol was tested on 5 PwMS and involved 18 sessions. The robot provided kinematic data to a real-time processor that interfaced with custom FES hardware and the display. FES has been shown to be effective in augmenting strength in stroke (Glinsky et al., 2007) and spinal cord injury (Martin et al., 2012) and in reducing motor fatigue in MS (Chang et al., 2011). Through advanced model-based controllers, the passive robotic arm support combined with FES was meant to improve movement quality by promoting accuracy and voluntary effort and to increase the intensity of the intervention with minimal therapist input. Promisingly, the results showed improved accuracy of tracking performance both when assisted and unassisted by FES, a reduction in the amount of FES needed to assist tracking, and a decreased impairment in the arm trained.

### 2.3 Weaknesses

#### 2.3.1 Challenges posed by MS symptoms

In Section 1, we have presented the classification of MS in the various subtypes and their associated symptoms. Among the five most common symptoms there are weakness/numbness in one or more limbs and visual impairments, such as painful monocular visual loss or double vision (Rolak, 2003). Both these symptoms result in a crucial weakness of robotic rehabilitation in PwMS. The robotic devices employed for PwMS rehabilitation are designed for unilateral use: when both limbs are affected and need to be trained, they have to undergo the training subsequently and not simultaneously. This procedure needs a longer duration of each rehabilitative session and requires moving the device and repositioning the user. A possible, but expensive and not always feasible, solution would be to synchronize two robotic devices to allow bimanual tasks and reduce downtime (Albanese et al., 2023). On the other side, visual impairments play a crucial role since all the studies about rehabilitation in PwMS (Carpinella et al., 2009; Feys et al., 2009; 2015; Vergaro et al., 2010; Basteris et al., 2011; Gijbels et al., 2011; Xydas and Louca, 2012; Octavia and Coninx, 2014; Sampson et al., 2016; Groppo et al., 2017; Maris et al., 2018; Solaro et al., 2020; Mannella et al., 2021; Tedesco Triccas et al., 2022) employed visual feedback as the main informative feedback to users. Indeed, these studies involved a virtual environment displayed on a screen and all of them required the absence of visual deficit as an eligibility criterion to participate in the study. The issue here is that visual impairments could affect subjects’ performance, impacting both their motor skills and their motivation. Despite being a weakness, robots allow adding other feedback, such as auditive or haptic, to replace or be integrated with the visual one to allow conducting the training. Finally, we need to take into account that 40%–70% of PwMS reveal cognitive symptoms, such as memory loss and dementia (DeLuca et al., 2015). As for visual impairments, most rehabilitative protocols are not meant to deal with cognitive deficits and require a minimum score in cognitive assessment tests as inclusion criteria. Among these tests, a Montreal Cognitive Assessment (MoCA) (Ng et al., 2015) score >15 was required by Manuli et al. (2020b) and a Mini-Mental (Pfeiffer, 1975) >24 by other studies (Carpinella et al., 2009; Gijbels et al., 2011; Gandolfi et al., 2018; Solaro et al., 2020).

#### 2.3.2 Limitations due to varied disease severity responses

Despite the potential benefits of robotic devices in the rehabilitation of neurological conditions (neurorehabilitation), their application is limited by difficulties in the integration between patient and machine, as well as by the variability of clinical conditions among patients, which may contribute to these difficulties. Guidelines offered by both Resquín et al. (2016) and Huang et al. (2017) emphasize the importance of careful patient selection criteria for robotic rehabilitation. For instance, among stroke patients, they suggest admitting only patients with moderate motor skills based on the Fugl-Meyer and Motor Assessment Scale scores. Previous studies indicate that robotic training is more beneficial for individuals with moderate to severe deficits, while those with better motor function do not experience greater benefits from innovative device training compared to conventional training (Duncan et al., 1983; Kim et al., 2010; Morone et al., 2011). Given this and the high variability in MS severity, the inclusion criteria employed by the studies of robotic rehabilitation for PwMS have requirements concerning minimal motor skills and minimal levels of disability. These criteria involved the Motricity Index (Collin and Wade, 1990) between 50 and 84 (Gijbels et al., 2011) or between 14 and 25 considering only the performance of the shoulder (Maris et al., 2018), the Expanded Disability Status Scale (EDSS) (Kurtzke, 1983) < 7.5 (Carpinella et al., 2009; Manuli et al., 2020b; Solaro et al., 2020) or between 1.5 and 8 (Gandolfi et al., 2018), the 9HPT (Kellor et al., 1971) score between 30 and 180 (Carpinella et al., 2009; Solaro et al., 2020) or 300 (Gandolfi et al., 2018) seconds.

#### 2.3.3 Device constraints and accessibility

There are several general considerations that need to be taken into account about the mechanical design of the devices used for robotic rehabilitation in PwMS. For instance, to simulate realistic daily actions, the devices must provide an adequate number of DoFs to allow for proper joint rotations. However, it is natural that some of the devices used in rehabilitation have limitations in this regard. For example, the Braccio di Ferro allows for elbow flexion/extension and shoulder internal/external rotations but only permits planar movement (Casadio et al., 2006). The Amadeo focuses on finger flexion/extension without allowing abduction and adduction (Gandolfi et al., 2018), while the Armeo Spring enables only forearm pronation and supination and is not intended to enable other wrist movements (Gijbels et al., 2011). Furthermore, although most of the devices presented in this context target the upper limb, they are designed to mobilize and measure only some of the upper limb joints. The Amadeo and Wristbot devices measure finger movements and wrist rotations, respectively, while other joints are not assessed and are held in place (Iandolo et al., 2019). The Braccio di Ferro and the Armeo Spring measure and control the shoulder and elbow joints, but the movement of the wrist (except for forearm pronation/supination in the Armeo Spring) is constrained. In contrast, the Haptic Master and the PHANTOM allow all the upper-limb joints to move freely, and the position of the end-effector determines their configuration and *vice versa* Feys et al. (2009, 2015), in the same way as it happens during usual interactions with objects. In addition to these constraints, both the affordability and accessibility of robotic devices should be considered in this analysis. While robotic-based rehabilitation has been shown to be effective, the cost and limited availability of these devices remain an issue. Despite being commercially available, robotic devices are not mass-produced, and their cost remains high (Laut et al., 2016). Considering PwMS frequent difficulties in walking and moving independently, it would be an added value to make robot-based rehabilitation more accessible, by setting such systems in undersupervised environments outside of treatment centers.

### 2.4 Opportunities

#### 2.4.1 General technological advancements

Robot-based MS rehabilitation will be definitely interested in the technological advancements that are currently involving the field of robotics. Rapid advances in digital electronics, hardware speed and accuracy, and fabrication techniques (Bezzo et al., 2015; Gopura et al., 2016) have played a crucial role in lowering the expenses associated with the production of both research prototypes and commercial products. The emergence of real-time control software, combined with the significant reduction in computing costs, has led to the development of a plethora of sophisticated and accurately controlled robotic devices for rehabilitation (Nizamis et al., 2021). Furthermore, advances in software, signal processing, and machine learning are expected to continue offering incremental contributions to technologies and algorithms for neurorehabilitation at an ever-increasing pace (Nizamis et al., 2021). The use of large amounts of data to implement machine-learning approaches could be facilitated by improving the overall networking between devices. Overall, the ongoing multidisciplinary approach is currently encouraging further synergies between traditional research in physics and engineering, chemical, biological, and medical science to develop new applications and acquire new capabilities.

#### 2.4.2 Promotion of digital health technologies

The goal of digital health is to improve healthcare outcomes, increase efficiency, and reduce healthcare costs by leveraging technology to optimize healthcare delivery and patient care. It encompasses a wide range of applications, including telemedicine, electronic health records, health data analytics, genomics, artificial intelligence, and mobile health apps. These technologies are being applied in various aspects of medicine, such as diagnosis, treatment, clinical decision support, care management, and care delivery (Mathews et al., 2019). Since the great promise held for improving healthcare outcomes, increasing efficiency, and reducing healthcare costs, the digital health sector has seen significant investment (Health, 2018). Digital health technologies provide a wealth of valuable data and connectivity, significantly amplifying the potential of robotics within healthcare. These technologies excel in the collection and storage of extensive patient data. By utilizing these vast datasets, it becomes feasible to identify critical trends and patterns, thereby strengthening research and the development of treatment strategies (Chang, 2023). Robotic systems can leverage this data to deliver personalized care, make real-time treatment adjustments, and facilitate monitoring, both in on-site and remote scenarios (Zhu et al., 2007; Barzilay and Wolf, 2013; Gross et al., 2013; Shirzad and Van der Loos, 2013). Advances in machine learning and deep learning have led to disruptive innovations in radiology, pathology, genomics, and other fields (Ramesh et al., 2004; MacEachern and Forkert, 2021). However, modern machine learning models require millions of parameters that need to be learned from sufficiently large, curated datasets to achieve clinical-grade accuracy while being safe, fair, equitable, and generalizing well to unseen data (Althnian et al., 2021; Varoquaux and Cheplygina, 2022). The issue here is how to address the problem of data governance and privacy by training algorithms collaboratively without exchanging the data itself. Rieke et al. (2020) proposed the approach of federated learning, which enables multiple parties to train collaboratively without the need to exchange or centralize data sets. Indeed, the machine learning processing occurs locally at each participating institution and only model characteristics are transferred. Concerning rehabilitation for PwMS, this approach has the potential to drastically increase sample size and enable a broader knowledge and an autonomous approach to the most correct practice to deal with the wide variety of symptoms of PwMS.

#### 2.4.3 The rapid surge of interest in home rehabilitation due to COVID-19

In the last decades, the demand for rehabilitation services has increased due to the aging phenomenon and the prevalence of chronic diseases, but it has faced a shortage of rehabilitation professionals and other barriers, such as low incomes, that deny access to rehabilitation services (Martinez-Martin and Cazorla, 2019). However, the continuity of exercises is crucial for the success of physical therapy and rehabilitation in PwMS, and the recommendation is to continue treatment practices in a home environment to support and maintain functional development in PwMS. Actually, at-home rehabilitation is suitable both for those requiring minor assistance and for those who cannot commute regularly to the treatment in physical therapy centers (Mayetin and Kucuk, 2022). Home-based rehabilitation has been found to be an effective alternative to traditional rehabilitation programs for stroke patients: it can be personalized and adjusted to the patient’s needs, can provide a more convenient and cost-effective therapy option (Catalan et al., 2018), achievable in a comfortable setting (Akbari et al., 2021). Additionally, unlike traditional exercises, where accountability relies on self-reporting, robotics enables objective monitoring, ensuring continuous exercise and program adherence. The COVID-19 pandemic caused an early discharge of existing patients, the suspension of new patients’ admissions, and the reduction of activities to decrease contacts (Manjunatha et al., 2021). Thus, the easiness of use and the low maintenance needed by haptic devices, together with the emerged necessity for further deploying the power of the internet for the purpose of communication and big data analysis, have attracted a lot of attention for home-based rehabilitation (Akbari et al., 2021). In particular, the need for effective home rehabilitation has received a boost from the need for early rehabilitative treatment to reduce long-term consequences in post-stroke patients (Akbari et al., 2021). Additionally, although many rehabilitation devices including robots are not available to be used at home due to their high costs, recent research has focused on low-cost devices able to assure both safety and effective results, in turn of a simpler design and functioning (Dowling et al., 2014; Rudd et al., 2019; Lambelet et al., 2020). The rehabilitation program for PwMS typically involves several sessions spread over a period of weeks or months. However, individuals with motor disabilities and limited mobility may face difficulties and expenses in traveling to health centers for treatment, which can pose a challenge in accessing rehabilitation services (Zasadzka et al., 2021). Even if this situation has been further complicated by COVID-19, the scenario might evolve into an opportunity for PwMS, as it happened for post-stroke patients. The future perspective is for an increase in the research interest in low-cost and easy-to-use robotic devices and in robot-based protocols for home-based rehabilitation in PwMS.

### 2.5 Threats

#### 2.5.1 Safety issues when dealing with spasticity

Spasticity is a common symptom in PwMS, characterized by an increase in the resistance of the joint to movement. This increased resistance is dependent on the velocity and caused by an amplification of the stretch reflex during the passive stretching of the joints (Trompetto et al., 2014). Therapeutic exercise treatments for individuals with high levels of spasticity can be challenging for therapists due to the patients’ considerable stiffness (Bohannon and Smith, 1987). This can create a barrier to the provision of sufficient rehabilitation therapy (Lee et al., 2017). Although most studies on robot-based rehabilitation in PwMS exclude individuals with spasticity (Carpinella et al., 2009; Sampson et al., 2016; Manuli et al., 2020b; Solaro et al., 2020), robots should be capable of addressing spasticity in a safe and appropriate manner. The proposed methods involve setting an appropriate torque range to manage spasticity-induced resistance or pausing movement when the resistance surpasses the motor’s threshold (Nam et al., 2017). The spastic muscle remains active for a certain amount of time with exponential decay of the resistance torque at the end of the range of motion where the robot is actuated against spasticity. This phenomenon allows the robot to be applied to a spastic limb even with a low-torque output motor without causing excessive loading. However, more precise sensing and control systems are necessary to deal safely with this symptom during PwMS rehabilitation.

#### 2.5.2 Risks related to unsupervised therapy

Robot-based rehabilitation is not meant to replace traditional therapies, but rather to complement them (Li et al., 2021). Robotic systems are ideal for providing intensive, task-oriented motor training for patients’ limbs under the supervision of a therapist, and are part of a suite of rehabilitation tools that includes nonrobotic methods (Harwin et al., 2006). However, the need to use healthcare resources efficiently is increasing, and there are proposals to enhance the cost-effectiveness of rehabilitation programs, including hospitals, care homes, and home-based rehabilitation (Ward et al., 2008). While robot-assisted therapy has typically been supervised by trained personnel, the trend towards home-based and autonomous rehabilitation is driven by the need to reduce staff workload, despite concerns about the deskilling of therapists and doctors (Li et al., 2021). By allowing patients to use robotics unsupervised or semi-supervised, therapists can handle multiple patients simultaneously during robot-assisted therapy sessions or allow them to receive robot-assisted training at home to increase therapy dose and regularity (Rosati et al., 2007; Ranzani et al., 2021). However, safety is a critical issue for minimally-supervised systems as they should be operated without supervision, restricting the role of the rehabilitation team to planning and remote monitoring (Rosati, 2010). Safety requirements necessitate that the robot does not move patients beyond their range of motion, avoids pressure points on fragile skin, and is easy to clean and compliant with infection control policies (Li et al., 2021). Despite concerns about safety and efficacy in the absence of qualified staff, recent research by Ranzani et al. (2021) has shown that a powered robot-assisted therapy device can be safely and intuitively used with minimal supervision by chronic stroke patients, while still meeting usability and perceived workload requirements. Interestingly, the study found that usability was inversely related to age but not to the level of impairment, with the oldest subjects experiencing the worst usability results (Ranzani et al., 2021). Given the high-dose therapy needed by PwMS, this population would definitely benefit from the increased dose and the continuum of care coming from home-based and autonomous rehabilitation, which could progressively increase patients’ involvement and autonomy from the clinic to home. While the potential for unsupervised therapies to complement conventional therapies in real-world settings is significant, safety and efficacy issues still need to be addressed to employ robotic devices with minimal supervision.

## 3 Virtual and extended reality in rehabilitation for PwMS

Previous sections listed the fields of a SWOT analysis for robotic rehabilitation systems tailored for PwMS. However, strengths can be empowered, opportunities can be exploited, weaknesses can be overcome, and threats can be prevented. Pondering the potential synergies of robotic devices with different technologies (as in the larger framework of Digital Health), virtual and augmented environments offer fertile solutions (De Angelis et al., 2021; Scholz et al., 2021; Kanzler et al., 2022a; Kanzler et al., 2022b; Chan et al., 2023).

### 3.1 Introduction to virtual and extended reality

Virtual Reality (VR) technologies can generate varying levels of immersion, defined as the sensation of being fully surrounded by a digital environment (Gandhi and Patel, 2018). Head-Mounted Displays (HMD) are typically used for full immersion, while large screens and projectors achieve semi-immersion, and simple monitors provide non-immersive VR experiences, even in common video games. At a psychological level, immersion can contribute to presence, the sensation of sharing time and space with another agent, object, or event through a medium. These processes, also enhanced through systems for haptic feedback and control, generally contribute to making the experience of a VR system engaging, especially when it is enriched by game-based features (Moline, 1997; Schuemie et al., 2001; Vafadar, 2013; Stone et al., 2014; Menin et al., 2018; Rose et al., 2018; Elor et al., 2020; Lee et al., 2020). However, VR constitutes just one category within a spectrum of environments, ranging from fully digital to entirely real, with some experiences involving no technological mediation in individual interactions. Originally, Milgram et al. (1995) defined the region between reality and VR as Mixed Reality (MR) to encompass all types of combinations of real and digital items. Within the sub-continuum of MR, Augmented Reality (AR) embraced the cases where digital items were perceptually inserted in a real scene, and Augmented Virtuality (AV) considered the situations where real objects enriched a digital setting. Additionally, different sub-types of AR emerged: overlay AR shows digital objects just floating in the visual scene, dissociated from the real context; encrusted AR represents digital objects visually behaving as real ones (they can be placed on a surface or they fall according to the law of gravity). An interesting case is the one of Spatial Augmented Reality (SAR) (Khademi et al., 2013), where the real environment is not augmented through a visor worn by a user but directly on its surfaces by means of screens or projectors. However, a debate on the meaning of such labels currently proceeds, leading to the adoption of the term Extended Reality (XR) as the set of combinations of digital and physical items and interactions (Stone, 2020). In all cases, the role of the digital items is to increase the information and the control opportunities offered by a User Interface (UI) or, specifically, a Graphical User Interface (GUI) (Carmigniani and Furht, 2011; Ejaz et al., 2019; Condino et al., 2022; Fu et al., 2022).

### 3.2 Virtual and extended environments for PwMS

Considering both virtual and augmented contexts, technologies represent a promising avenue for rehabilitation interventions. Indeed, they offer immersive and interactive experiences that enhance patient motivation and treatment outcomes (Khademi et al., 2013; Regenbrecht et al., 2014; Mubin et al., 2019; Sánchez-Herrera-Baeza et al., 2020; Leong et al., 2022; Araujo et al., 2023), including cases of MS (Massetti et al., 2016; Jonsdottir et al., 2019; Kalron et al., 2022; 2020; Cuesta-Gómez et al., 2020; Chadali et al., 2023; Milewska-Jędrzejczak and Głąbiński, 2023). Indeed, VR and AR environments have proven to encourage neuroplasticity in neurological patients in terms of learning abilities and verbal short-term memories, as well as improve fatigue and quality of life (Huang et al., 2022; Araujo et al., 2023; Milewska-Jędrzejczak and Głąbiński, 2023). It must also be noted how, due to its impact on their vestibular systems, VR seems to have specific effects on PwMS, altering their sense of presence and even leading to discomfort and cybersickness in immersive environments. This observation highlights the need for designing multimodal feedback solutions to enhance the accessibility of interactive settings (Guo and Quarles, 2012; Samaraweera et al., 2015; Arafat et al., 2016; Mahmud et al., 2023; 2022; Hollywood et al., 2022). In a study conducted on 54 PwMS undergoing a 12-week VR-based rehabilitative intervention, Saladino et al. (2023) showed the effectiveness of such an approach in improving abilities in performing activities of daily living, quality of life, and satisfaction throughout the therapeutic sessions. Overall, it is worth highlighting that when comparing VR *versus* XR technologies in rehabilitation, the latter sees way less employment with respect to the first one (Mubin et al., 2019). A rare example is the study of Pruszyńska et al. (2022) which assessed the effects of applying AR in telerehabilitation for PwMS over a 4-week experimental study. Although no particular difference has been found in neuroplasticity between AR and conventional therapy groups, a significant decrease in task execution time and an increase in grip strength with respect to the control group was noted in both arms.

Furthermore, through the use of serious games (games devised for non-leisure applications too, from education to therapy) and the approach of gamification (adding game features to non-leisure systems), the patient is led to perform repetitive training activities with higher motivation and, consequently, clinical adherence (Godfrey and Barresi, 2022). VR-based exergames have garnered attention within MS rehabilitation, particularly for targeting upper limb movements (Webster et al., 2021; Pau et al., 2023b; Chadali et al., 2023). These games combine task-oriented exercises with elements of gamification to create engaging rehabilitation experiences (Jonsdottir et al., 2019). They offer diverse exercise scenarios, allowing customization based on individual patient requirements and variations in symptoms (Leocani et al., 2007). Additionally, they can offer options for patients who could refuse fully immersive settings or excessively playful ones. VR/XR exergames can provide real-time feedback and visual cues, enhancing patient immersion and performance (Hsu et al., 2023) and serving as tools for self-guidance and self-evaluation (Sousa et al., 2016; Bucchieri et al., 2022). Indeed, it has been shown that providing appropriate feedback to the user is crucial to enhance movement correctness, directly linked to therapy effectiveness (Cavalcante Neto et al., 2020). However, it is worth noting that many studies in the literature rely on commercially available exergames, which are often not customizable (Webster et al., 2021).

Virtual settings can offer an immersive experience and a sensation of presence to empower the individual motivation in repetitive tasks that can be psychologically and physically tiresome even if the patient interacts with a robotic device (Tang et al., 2005; Clark et al., 2019). On the other hand, different from robotic rehabilitative systems present in clinics, VR/XR devices represent an optimal solution for home-based rehabilitation. Cost-effective and easy to set up, they can provide useful settings for occupational therapy (Corrêa et al., 2013; Pruszyńska et al., 2022; Tada et al., 2022). An example is the immersive virtual kitchen game proposed by Pau et al. (2023a) where the environment provides several activities of daily living tasks (e.g., tidying up, cooking, washing the dishes, *etc.*). Questionnaires conducted on an 8-week study on PwMS showed overall great appreciation and satisfaction with the proposed rehabilitative environment.

Another aspect of such systems is the presence of refined sensors to collect kinematic measurements (i.e., upper-limb and ocular movements) and quantify the quality of patients’ recovery (Leocani et al., 2007; Pillai et al., 2022; Tada et al., 2022; Herrera et al., 2023). Soares et al. (2021) evaluated the hand tracking accuracy of HTC Vive (immersive VR headset) and HoloLens2 (XR headset) with respect to a Motion Capture (MoCap) system, while Pascucci et al. (2022) and Pillai et al. (2022) compared the use of a Microsoft Kinect-based prototype and HoloLens2. These studies revealed the suitability of VR and XR headsets to detect jerky behavior in kinematic data and unexpected hand movements, and to precisely evaluate movement tracking. Nonetheless, with such devices, it might be possible to perform accurate rehabilitative assessments and evaluate motor dysfunctions in PwMS (Herrera et al., 2023). Although such a feature might appear redundant when using a robotic system, the data obtained through VR/XR devices can be integrated with the information collected by the robotic platforms. This integration creates a more comprehensive and enriched portfolio of the therapeutic journey, enhancing the overall experience and outcomes.

## 4 Robotic and VR integration for MS rehabilitation

### 4.1 Mechatronic-digital synergies

In this manuscript, we analyzed how robot-assisted rehabilitation for PwMS has the potential to represent a significant advancement. Additionally, especially when integrated with virtual or augmented environments, this approach can offer an even more patient-centric solution that goes beyond the limitations of standard care.

Previous studies have emphasized the potential of combining robotic and digital technologies for rehabilitation, highlighting the benefits of synergistic strategies. Patton et al. (2006a) remarked how the flexibility of both approaches can offer promising solutions for cost-effective, time-efficient, and repetitive exercises for neurological (specifically, brain-injured) patients. These solutions exploit our knowledge of the nervous system with the versatility of virtual/augmented settings and the accuracy and precision of robots, opening up exciting possibilities. Achieving success in this field depends on the active involvement of therapists and clinicians in the co-design process, as well as the state-of-the-art in haptic and graphic systems. The latter point is particularly interesting, as demonstrated by recent studies by Atashzar et al. (2019), highlighting the need for matching the patients’ needs and biomechanics, the stability in physical patient-robot interaction with a high level of fidelity of force field, the chance of implementing home solutions for improving clinical outcomes while reducing adoption costs.

Overall, the debate on VR-enhanced motor-cognitive rehabilitation robotics (Riener et al., 2006) always focused on the capability of multimodal displays to make therapy more exciting and increase patient engagement. Indeed, visuo-haptic approaches derived from the synergy of digital and mechatronic solutions were definitely impactful for engaging subjects through “reality distortion”, by using error augmentation strategies (Patton et al., 2006b). Mutually, the haptic properties in human-robot interaction can enrich visual digital items with tactile features that raise up user engagement (D’Antonio et al., 2021; Frisoli et al., 2009; Gueye et al., 2021; Li et al., 2014; Ruffaldi et al., 2014). These were just exemplary studies on the documented principles underlying the synergies of virtual and robotic systems in rehabilitation, and the literature reveals an even richer set of investigations in this area (Qiu et al., 2010; Guidali et al., 2011; Merians et al., 2011; Cortés et al., 2014; Russo et al., 2018; Mubin et al., 2019; Manuli et al., 2020a; Torrisi et al., 2021).

Notably, Zanatta et al., 2023a, Zanatta et al., 2023b, Zanatta et al., 2022) explored the potential of combining robotic and virtual systems in rehabilitation, by analyzing their practical implications. The authors proposed a biopsychological approach to examine the points of view offered by multiple health-related perspectives, according to diverse technological solutions and heterogeneous conditions. Through such a vision, especially useful in Health Technology Assessment (HTA), they addressed public health challenges and healthcare sustainability for evaluating the introduction of the aforementioned integration of digital and mechatronic systems in rehabilitation. In particular, addressing the limitations reported by patients is essential. Patients generally reported high levels of acceptance, satisfaction, and perceived safety during treatments involving the combined use of these technologies. Nevertheless, the authors emphasized the need for in-depth advances in terms of assessment: the next subsection proposes an approach for handling this challenge, with a special focus on the context of PwMS upper-limb rehabilitation.

### 4.2 Towards an integrative SWOT analysis

To develop a comprehensive and fully functional rehabilitation platform beneficial for PwMS, the two technological domains of robotics and virtual/extended reality must address their respective weaknesses while leveraging their strengths in rehabilitation. Utilizing embedded sensors, robots and VR/XR technology can create dynamic environments that promote sensorimotor restoration. For example, headsets can immerse individuals in a digital world, enabling them to engage in tasks closely resembling real-life challenges. However, interacting solely with virtual objects may lead to a loss of the sense of reality. Robotic devices, on the other hand, can provide haptic feedback and generate a strong sense of involvement in the task. For instance, the use of devices like the Haptic Master in conjunction with task-specific and engaging non-immersive virtual environments, such as the I-TRAVLE system (Maris et al., 2018), has demonstrated promising results in PwMS rehabilitation.

Selecting the appropriate rehabilitative strategy becomes particularly challenging when dealing with neurological disorders. PwMS often exhibit unique symptoms, leading to the need for optimal robotic control strategies and visual stimuli to provide personalized assistance. From a kinematic impairment perspective, PwMS may experience varying degrees of disability in both arms, with the more affected side not necessarily corresponding to the dominant one. However, given the main focus of the researchers on stroke-related impairments, the majority of upper-limb exoskeletons are designed for unilateral use. To address this limitation, immersive VR/XR technologies can promote bi-manual exercises and accurately measure the user’s hand movements in the non-treated arm, creating a comprehensive digital representation of the patient. This clinical portfolio can provide valuable quantitative data for monitoring the rehabilitation progress and training predictive AI models. Alternatively, when dealing with cognitive impairments, VR/XR environments can be simplified to make tasks more accessible and easier to understand or enriched with challenging mental exercises to enhance neuroplasticity.

VR systems, characterized by their ease of access and cost-effectiveness, present also an attractive option for home-based rehabilitation, complementing clinic-based robotic interventions and assuring the continuum of care needed by PwMS. In cases of relapse or hospitalization, robot-assisted rehabilitation often takes precedence. During this phase, patients may require passive movement, extensive assistance, and gravity compensation, which can be facilitated through robotic devices. However, once this phase concludes, the challenge lies in the paucity of robotic devices suitable for home-based rehabilitation. To ensure continuous rehabilitation and patient monitoring, VR systems, enhanced by computer vision techniques, can be employed by patients in their own homes. This combination allows patients to continue their rehabilitation at home, preventing issues like de-conditioning, muscle weakness due to limited mobility, and muscle contractures associated with spasticity. This synergy offers a holistic approach that keeps patients trained, engaged, and tracked in their recovery journey. This synergistic approach fosters a comprehensive strategy that not only keeps patients actively engaged but also facilitates effective tracking throughout their recovery journey. Importantly, relying on VR for home-based rehabilitation carries fewer risks associated with unsupervised therapy. While VR-based rehabilitation encourages active movements, its limitation in haptic feedback diminishes potential adverse effects, thereby enhancing safety during unsupervised sessions. This innovative integration not only propels the efficacy of home-based rehabilitation but also underscores a commitment to patient wellbeing and progress.

**FIGURE 2:**
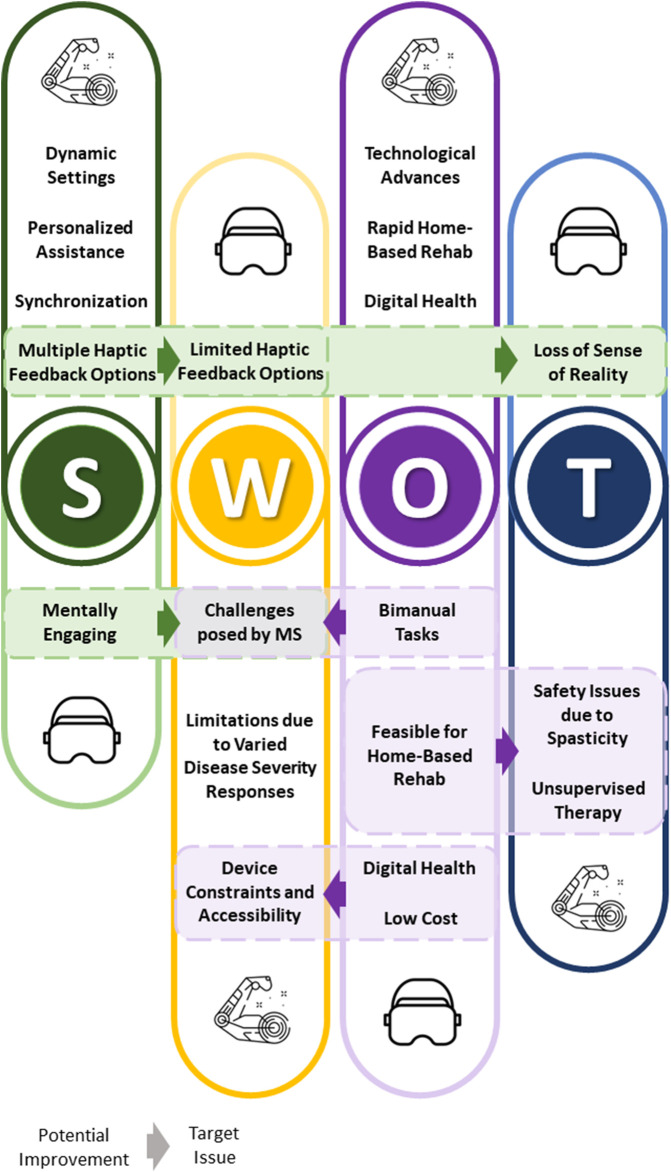
This figure summarizes a unified SWOT analysis for robotics and VR, along with their interrelated elements. Dashed boxes contain strengths or opportunities of VR that can help overcome the weaknesses and threats of robotic devices, and *vice versa* as discussed in Section 4. The direction of the arrows indicates which potential improvement (stemming from strengths or opportunities) of VR or robotic devices could address a target issue (weakness or threat) of the other technology.

Figure 2 provides a holistic overview of a comprehensive SWOT analysis that includes both robotics and VR. This visual representation illustrates how the advantages of each of these two innovative technologies can be exploited to complement each other and overcome their respective limitations. To sum up, considering the SWOT analysis of robotic systems performed above, the synergy with virtual and augmented environments can:

• Enhance the strengths observed about dynamic environments (generating engaging contents), haptic feedback (through its visual and multimodal counterparts), personalized assistance (stimulating the person with a novel game-like scenario for the same robot-based task when the user is tired, as in the Cypress approach);
• Mitigate the weaknesses in accessibility by presenting an ecologically valid and intuitive setting for rehabilitation, and the weaknesses related to the challenges posed by MS by creating a more comprehensive digital representation of the patient;
• Develop the opportunities offered by Digital Health and telerehabilitation, especially considering how low-cost technologies for VR and XR are proposed at a high pace on the market;
• Prevent the safety issues related to the rise of unpredictable symptoms (e.g., spasticity) and the possible consequent threats during unsupervised therapy.

## 5 Conclusion

Our review focused on the use of robotic systems in the context of Multiple Sclerosis rehabilitation, with a particular emphasis on conducting a SWOT analysis of these systems. Our comprehensive goal was to assess whether the synergy with other systems, in this case virtual and extended reality (VR/XR) technologies, could enhance the Strengths, mitigate the Weaknesses, explore new Opportunities, and address the Threats of rehabilitation robotics for MS. We found that both robotic systems and VR/XR technologies exhibit distinct advantages and disadvantages. Some of these limitations cannot be entirely overcome by combining the two technologies, yet there are clear areas where their synergy can be highly beneficial. Although providing haptic feedback through robotic devices remains critical especially when MS symptoms are severe, the incorporation of VR systems, offers clear advantages for home-based rehabilitation, aligning with the growing need for a continuum of care in the management of MS. Moreover, the integration of VR/XR technologies can enhance engagement and realism in rehabilitation tasks, making them more closely resemble activities of daily living (ADL). It is noteworthy that we chose to primarily focus on VR/XR technologies due to their widespread use, simplicity, and ease of integration into the existing healthcare landscape. While other technologies, such as Functional Electrical Stimulation (FES) or EEG-based brain–computer interfaces (Said et al., 2022), hold promise, we believe that starting with VR is a pragmatic approach given its established presence and adaptability within the current context. In summary, our analysis provides an in-depth perspective on the current framework within which robotic technologies are integrated into Multiple Sclerosis MS rehabilitation. Moreover, it underscores the transformative impact of merging robotic systems with VR technologies, shedding light on their individual strengths and areas for enhancement. This synthesis paves the way for a promising future, aiming to deliver more effective and accessible rehabilitation solutions for individuals facing the challenges of MS.
